# Chemical Profiles and Insecticidal Potential of Essential Oils Isolated from Four *Thymus* Species against *Rhyzopertha dominica* (F.)

**DOI:** 10.3390/plants11121567

**Published:** 2022-06-14

**Authors:** Asgar Ebadollahi, Bahram Naseri, Zahra Abedi, William N. Setzer

**Affiliations:** 1 Department of Plant Sciences, Moghan College of Agriculture and Natural Resources, University of Mohaghegh Ardabili, Ardabil 5697194781, Iran; 2 Department of Plant Protection, Faculty of Agriculture and Natural Resources, University of Mohaghegh Ardabili, Ardabil 5697194781, Iran; bnaseri@uma.ac.ir (B.N.); zahra.abedi@uma.ac.ir (Z.A.); 3 Aromatic Plant Research Center, 230 N 1200 E, Suite 100, Lehi, UT 84043, USA

**Keywords:** essential oil, biochemical disruption, nutritional indices, *Rhyzopertha dominica*, *Thymus*, toxicity

## Abstract

Although chemical pesticides have been efficiently used to manage insect pest, their overuse has led to environmental contamination and threats to human health, enticing researchers to introduce eco-friendly and effective agents. In this study, the insecticidal effectiveness of essential oils isolated from *Thymus* species, including *T. eriocalyx*, *T. kotschyanus*, *T. fallax*, and *T. vulgaris*, was evaluated against the adults of *Rhyzopertha dominica*. The terpenes *p*-cymene, 1,8-cineole, linalool, α-terpineol, and carvacrol were the prominent compounds in the hydrodistilled essential oils. All essential oils produced significant fumigant at 24, 48, and 72-exposure times. The energy reserves protein by all essential oils, glycogen by *T. kotschyanus* and *T. vulgaris*, and lipid by *T. fallax* and *T. vulgaris* were significantly decreased compared to control. All essential oils except *T. vulgaris* affected the amylolytic and proteolytic activity of the pest. The pest increased the α- and β-esterase enzyme activity in response to the essential oils. Nutritional indices of adults were also affected by essential oils, in which feeding deterrence index was calculated from 20.41% to 61.11%. Accordingly, based on lethal and extensive sub-lethal insecticidal activities, *T. eriocalyx*, *T. kotschyanus*, *T. fallax*, and *T. vulgaris* essential oils can be considered as efficient agents for *R. dominica* management.

## 1. Introduction

The lesser grain borer, *Rhyzopertha dominica* (F.) (Coleoptera: Bostrichidae), is one of the damaging primary insect pests of stored grains especially wheat and rice throughout most of the world [1,2]. The adults have high viability potential and are strong flyers that can find the hosts from up to 1 km distance [3]. *Rhyzopertha dominica* is an internal feeder, and the whole life of larvae and most of the adult life, as harmful stages of the pest, are spend inside a single kernel [4]. As fumigants can penetrate areas where insects hide, fumigation of an active substance is the main strategy in the management of stored-product insect pests [5]. Although synthetic fumigants such as phosphine (PH_3_) have a very good penetrant feature and quick lethality actions, frequent application of these chemicals has resulted in several side effects including environmental contamination, threat to human health and non-target organisms, and developing insect pest resistance [6,7].

The promising insecticidal effects of plant-derived essential oils fumigated on several stored product insect pests have been reported [8,9,10]. Recently, the insecticidal activities of essential oils against *R. dominica* were reported: The significant fumigant toxicity of essential oil extracted from *Satureja intermedia* C. A. Mey was revealed against the adults of *R. dominica* which positively depended on the concentration of the essential oil and the exposure times [11]. In another study, along with considerable lethality, the fumigation with *Anethum graveolens* L. and *Melaleuca cajuputi* Powell essential oils resulted in significant reduction in total protein and detoxifying enzymes (esterases and glutathione-*S*-transferase) activity of *R. dominica* adults [12]. According to the study of Tine et al. [13], *R. dominica* adults were susceptible to the essential oil of *Lavandula angustifolia* L., in which along with fumigant toxicity, the essential oil decreased α-amylase and protease activities of treated adults. Accordingly, it is possible to control *R. dominica* using fumigation of essential oils.

The plant species from the genus *Thymus* L. (Lamiaceae) are among the most popular aromatic plants throughout the world based on their medicinal and food additive properties [14]. *Thymus* are also known for dense shrubs, robust root system, small leaves, pleasant aroma and flavor, and high amount of essential oil [15], which are commonly used as food preservatives and cosmetic procurements [16]. Insecticidal effects of essential oils isolated from some *Thymus* species against stored product insect pests was documented. For example, fumigant toxicity of *Thymus persicus* (Ronniger ex Rech. f.) Jalas essential oil, with carvacrol (44.69%) and thymol (11.05%) as main compounds, against the adults of red flour beetle (*Tribolium castaneum* Herbst) and the rice weevil (*Sitophilus oryzae* (L.)) was reported by Saroukolai et al. [17]. Fumigant toxicity of *Thymus pallescens* Noë. essential oil, with carvacrol (56.64%), *p*-cymene (16.36%), and thymol (8.71%) as main compounds, against the granary weevil (*Sitophilus granarius* L.) was found by Moutassem et al. [18]. Along with lethality, the *T. pallescens* essential oil decreased total protein, lipid, and carbohydrate of treated weevil. In another study, fumigation with *Thymus quinquecostatus* Celak essential oil and its major components linalool (52.00%), borneol (10.91%), and anethole (5.33%) resulted in significant mortality of the cigarette beetle (*Lasioderma serricorne* (F.)) and *T. castaneum* adults [19].

As part of our continuing search, assessment of chemical profiles and insecticidal effects of essential oils obtained from four *Thymus* species *T. eriocalyx* (Ronniger) Jalas, *T. kotschyanus* Boiss. & Hohen, *T. fallax* Fisch. & C.A. Mey, and *T. vulgaris* L. against the adults of *R. dominica* was the main objective of this study. In the insecticidal assays, along with fumigant toxicity, the biochemical disruptions in energy resources content and esterase, amylase, and protease enzyme activities, and antinutritional activity of essential oils on the treated adult insects were also assessed.

## 2. Results

### 2.1. Chemical Profile of Essential Oils

The major components in the *T. eriocalyx*, *T. fallax*, *T. kotschyanus*, and *T. vulgaris* essential oils were *p*-cymene (4.8%, 7.5%, 2.2%, and 3.4%, respectively), 1,8-cineole (7.4%, 5.4%, 5.7%, and 7.6%), linalool (3.2%, 15.4%, 9.5%, and 4.1%), α-terpineol (4.8%, 4.7%, 11.4%, and 7.7%), carvacrol (19.3%, 2.4%, 1.6%, and 9.7%), and α-terpinyl acetate (4.0%, 6.3%, 18.9%, and 0.2%). Interestingly, thymol was abundant in *T. eriocalyx* (8.6%), *T. fallax* (12.1%), and *T. vulgaris* (9.6%), but was not observed in *T. kotschyanus*. Limonene was an abundant constituent of *T. kotschyanus* (5.3%), however. A high concentration of borneol was observed for *T. eriocalyx* (5.7%). A relatively high concentration of nerol was found in *T. kotschyanus* (5.6%) and *T. vulgaris* (4.3%) essential oils, while geraniol was abundant in *T. vulgaris* (18.7%). Neryl acetate was also relatively abundant in *T. vulgaris* essential oil (6.3%). Although essential oils were rich in different groups of terpenic compounds, especially *T. eriocalyx* essential oil 100% composed of terpenes, phenylpropanoids were also identified in *T. fallax* (0.1%), *T. kotschyanus* (7.9%), and *T. vulgaris* (4.2%) essential oils (Table 1).

### 2.2. Fumigant Toxicity

Data of the mortality of *R. dominica* adults treated by the essential oils of *T. eriocalyx* (Z = 0.853: 2-tailed Significant = 0.462), *T. fallax* (Z = 0.979: 2-tailed Significant = 0.293), *T. kotschyanus* (Z = 1.370: 2-tailed Significant = 0.047), and *T. vulgaris* (Z = 1.348: 2-tailed Significant = 0.053) had statistically normal distributions based on the results of Kolmogorov-Smirnov test. Analysis of variance indicated that concentrations of *T. eriocalyx* (F = 264.0)*, T. fallax* (F = 119.5), *T. kotschyanus* (F = 145.3), and *T. vulgaris* (F = 77.00) essential oils had significant effects on the insect mortality (df = 5, 36: *p* < 0.001). The exposure time from 24 to 72 h of *T. eriocalyx* essential oil resulted in significant mortality of the insect (F = 3.733: df = 2, 36: *p* = 0.034), while effects of exposure time of other essential oils along with the interaction of essential oil concentration with time were not significant.

Probit analysis of data obtained from the fumigant toxicity of *Thymus* essential oils against *R. dominica* adults revealed that a concentration of 180.9, 157.0, 174.5, and 174.7 µL/L, respectively, from *T. eriocalyx, T. fallax, T. kotschyanus*, and *T. vulgaris* essential oils was adequate to achieve 90% mortality of tested insects (LC_90_ values) after 72 h. According to the high *r*^2^ values accessible in Table 2, there is a positive direct relation between tested concentrations of *Thymus* essential oils and the mortality of *R. dominica*. Although the calculated LC_50_ values are close to each other, the essential oil of *T. kotschyanus* with the lowest LC_50_ and the highest relative potency at all exposure times showed higher toxicity than others (Table 2). On the other hand, the adults of *R. dominica* were more susceptible to the *T. kotschyanus* essential oil than the other species. Calculated 24-h LC_30_ values, including 84.16, 85.36, 75.57, and 103.23 µL/L for *T. eriocalyx, T. fallax, T. kotschyanus,* and *T. vulgaris* essential oils, respectively, were selected for sublethal experiments.

### 2.3. Effect of Essential Oils on Energy Reserves

Variations in protein, glycogen and lipid content of *R. dominica* adults treated by LC_30_ and LC_50_ of *T. eriocalyx, T. fallax*, *T. kotschyanus*, *and T. vulgaris* essential oils are shown in Table 3. The LC_30_ values had no significant effect on the protein, glycogen and lipid content, but with increasing concentration to LC_50_, the content of these macromolecules decreased. The treatment of adults with LC_50_ values of all tested essential oils significantly reduced their total protein content compared to the control (*p* < 0.05). Although the glycogen content of adults decreased by all essential oils, only the reduction affected by *T. kotschyanus* and *T. vulgaris* essential oils was significant compared to the control group (*p* < 0.05). Regarding lipid content, the more decrease delivered by *T. fallax* essential oil, followed by *T. eriocalyx* and *T. vulgaris* essential oils (Table 3).

### 2.4. Effect of Essential Oils on Esterase Activity

Esterase enzyme activity of *R. dominica* adults treated with LC_30_ and LC_50_ of *T. eriocalyx, T. fallax, T. kotschyanus,* and *T. vulgaris* essential oils was significantly increased compared to the control group (*p* < 0.05) (Table 4). The activity of α-esterase enzyme by *T. kotschyanus* and *T. vulgaris* treatment was higher than *T. eriocalyx* and *T. fallax*. The β-esterase activity in the adults treated by the essential oils of *T. fallax* and *T. vulgaris* had greater increases than *T. eriocalyx* and *T. kotschyanus* (Table 4).

### 2.5. Effect of Essential Oils on Amylolytic and Proteolytic Activity

Although the LC_30_ of essential oils had no considerable effect on the amylase and protease activities, treatment of *R*. *dominica* adults with LC_50_ values of *T*. *eriocalyx*, *T*. *kotschyanus* and *T. fallax* essential oils significantly decreased the activity of these digestive enzymes (*p* < 0.05). The effect of *T. vulgaris* essential oil on the activity of amylase and protease enzymes was not significantly different compared to the control (*p* < 0.05). Essential oils of *T*. *kotschyanus* and *T. fallax* caused the most decrease in amylase enzyme activity. *T*. *kotschyanus* essential oil also reduced the protease enzyme activity more than other essential oils (Table 5).

### 2.6. Effect of Essential Oils on Nutritional Indices

The effects of LC_30_ and LC_50_ values of *Thymus* essential oils on nutritional indices of *R. dominica* adults are shown in Table 6. The consumption index (CI) of adults treated by both LC_30_ and LC_50_ of *T. eriocalyx*, *T. kotschyanus*, and *T. vulgaris* essential oils was significantly reduced compared to the untreated insects (*p* < 0.05). Both LC_30_ and LC_50_ of *T. fallax* essential oil had no significant effect on CI of adults. The efficiency of conversion of ingested food (ECI) was significantly decreased by LC_30_ and LC_50_ of *T. fallax* and *T. kotschyanus* (*p* < 0.05). Treatment by LC_30_ and LC_50_ values of *T. eriocalyx*, *T. kotschyanus*, and *T. vulgaris* essential oils resulted in a significant reduction in the relative consumption rate (RCR) of *R. dominica* adults (*p* < 0.05). While the LC_30_ of *T. fallax* essential oil had no significant effect on the RCR, it was significantly reduced by the LC_50_ value compared to the control (*p* < 0.05). Although tested concentrations of all essential oils significantly decreased the relative growth rate (RGR) (*p* < 0.05), the lowest RGR was observed in the adults treated with *T. kotschyanus* essential oil. The essential oils of *T. vulgaris* and *T. kotschyanus* showed the highest feeding deterrence index (FDI), with both LC_30_ and LC_50_, followed by *T. eriocalyx* and *T. fallax* oils (Table 6).

## 3. Discussion

**Figure 1:**
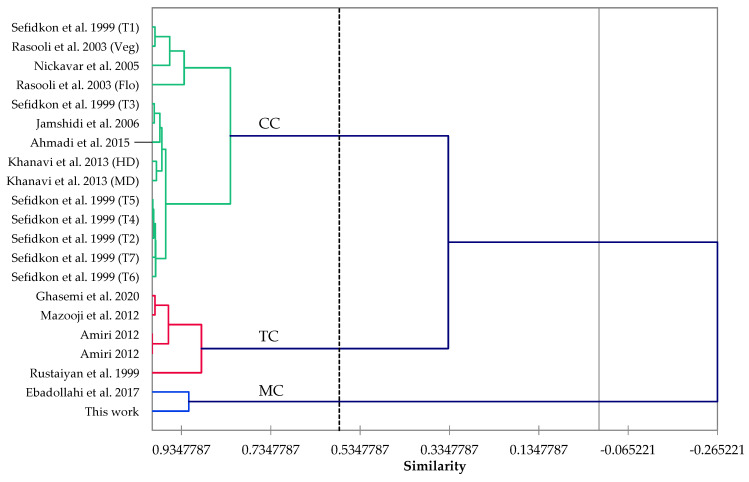
Dendrogram obtained by cluster analysis of the percentage composition of essential oils from *Thymus kotschyanus* samples, based on the correlation and using the unweighted pair-group method with arithmetic average (UPGMA). CC: Carvacrol chemotype. TC: Thymol chemotype and MC: Mixed (α-terpinyl acetate/α-terpineol/linalyl acetate/linalool) chemotype. Amiri 2012 [26], Sefidkon et al. 1999 [36], Khanavi et al. 2013 [37], Rasooli et al. 2003 [38], Nickavar et al. 2005 [39], Jamshidi et al. 2006 [40], Ahmadi et al. 2015 [41], Ghasemi et al. 2020 [42], Mazooji et al. 2012 [43], Rustaiyan et al. 1999 [44], Ebadollahi et al. 2017 [45].

Chemical composition of *T. eriocalyx*, *T. fallax*, *T. kotschyanus*, and *T. vulgaris* essential oils was investigated in some recent studies. An extensive examination of *T. eriocalyx* essential oils from different locations in Iran showed a high degree of variation in the essential oil compositions [23,24], as observed in other countries on *Thymus* and other genera of Lamiaceae family [25]. They described three chemotypes, (1) a linalool chemotype, (2) a thymol chemotype, and (3) a geraniol chemotype. The *T. eriocalyx* essential oil in this work represents a chemotype that may be the best described as a thymol/carvacrol mixed chemotype, more closely resembling sample T2 from Sefidkon et al. [24] and a *T. eriocalyx* sample analyzed by Amiri [26]. Based on previous reports on *T. fallax* essential oil compositions, there are two general chemotypes: (1) a thymol-rich chemotype and (2) a carvacrol-rich chemotype. However, The *T. fallax* essential oil sample in this study does not fit with either of those two chemotypes; both thymol (12.1%), and carvacrol (2.4%) concentrations were low, and this essential oil sample may represent a new chemotype, rich in linalool [27,28,29,30]. Based on the essential oil composition, agglomerative hierarchical cluster analysis has revealed that three chemotypes of *T. kotschyanus* can be defined: (1) a carvacrol-rich chemotype (>80% similarity within samples), (2) a thymol-rich chemotype (around 90% similarity within samples), and (3) a mixed chemical type dominated by α-terpinyl acetate, α-terpineol, linalyl acetate, and linalool, which includes the essential oil in this work (see Figure 1) and is completely dissimilar to the carvacrol and thymol chemotypes. An analysis of numerous *T. vulgaris* essential oils reported in the literature had revealed four major chemotypes, (1) a thymol-rich chemotype, (2) a carvacrol-rich chemotype, (3) a linalool-rich chemotype, and (4) a geraniol-rich chemotype [31]. Most *T. vulgaris* samples from Iran fall into the thymol-rich chemotype [32,33,34,35]. However, the *T. vulgaris* essential oil in this study belongs to a geraniol-rich chemotype. It can also be noted that differences in exogenous and endogenous factors affecting the chemical profiles of *Thymus* essential oils, including geographical location, genetic make-up and growth stages of plants, and extraction method [24,31,36,37], have resulted in the above-mentioned differences.

The insecticidal efficiency of essential oils was related to their compounds [9,46]. Rozman et al. [47] showed that the monoterpenes 1,8-cineole, carvacrol, and linalool occurring in *T. vulgaris* essential oil, and recognized as main compounds of all essential oil studied in our study, had promising fumigant toxicity against the adults of *R. dominica*. Fumigant toxicity of *p*-cymene and α-terpineol, the other main compounds in the present study, against the adults of *R. dominica* was also demonstrated by Shaaya et al. [48] and Filomeno et al. [49]. Rozman et al. [47] also concluded that linalool was highly effective than 1,8-Cineole and carvacrol. Although linalool was identified in *T. eriocalyx* (3.2%) and *T. vulgaris* (4.1%) essential oils, its level was higher in the essential oils of *T. fallax* (15.4%), *T. kotschyanus* (9.5%). Furthermore, according to the study of Rozman et al. [47], linalyl acetate had significant fumigant toxicity against *R. dominica* adults, which was recognized in both *T. fallax* (0.6%) and *T. kotschyanus* (7.9%) essential oils, and these can justify the toxicity of *T. fallax* and *T. kotschyanus* essential oils, which were higher than *T. eriocalyx* and *T. vulgaris*. 

Reducing the amount of energy resources the proteins, carbohydrates, and lipids considered one of the main strategies in the insect pest management because of their important roles in insect biochemical pathways, growth, metamorphosis, reproduction, and diapause [50,51,52]. On the other hand, changes in energy reserves specify the susceptibility of the insect to tested materials [53]. In this study, though reductions in glycogen content by *T. eriocalyx* and *T. fallax* essential oils and in lipid content by *T. kotschyanus* essential oil were not significantly different than controls (*p* < 0.05), the energy content of *R. dominica* adults in other treatments was reduced. These results agreed with Yazdani et al. [54], Jyoti et al. [55], and Moutassem et al. [18] who discovered the reduction in protein, carbohydrate, and lipid contents of insect pests treated with *Thymus* essential oils. Insects normally are able to convert carbohydrates into lipids [53], which could explain the relatively lower reduction in lipid content due to the essential oil of *T. kotschyanus* compared to other essential oils.

Exposure to lethal and sublethal concentrations considerably affected the enzymatic activities of an organism, reflecting the biochemical disturbances [56]. Hence, to clarify the mechanism responsible for the toxicity of *Thymus* essential oils, effects on digestive amylase and protease enzymes and detoxifying α- and β-esterase activities were also studied. In this study, the activity of digestive enzymes amylase and protease was reduced in *R*. *dominica* adults treated by *T*. *eriocalyx*, *T*. *kotschyanus*, and *T. fallax* essential oils. Similar supportive results have been reported in the case of reducing the digestive enzyme activities in insect pests treated by essential oils [57,58,59], showing strong insecticidal action. It may be due to cytotoxic effects on epithelial cells of alimentary canal which reduced the secretion rate of their enzymes [52,59].

Treatments with the LC_30_ and LC_50_ of *T. eriocalyx, T. fallax, T. kotschyanus,* and *T. vulgaris* essential oils significantly increased the activity of the α- and β-esterases compared to control. The esterases can hydrolyze the ester bonds in a toxic substance and detoxify them in the insect’s body [60]. The high activity of esterase enzymes indicates that the insect’s defense mechanisms are active against the treating agents and can be consider as development of resistance [61,62]. In general, regardless of the type of influencing exogenous factor, the insect activates its immune or defense mechanisms, such as increasing detoxifying enzymes, which will require significant energy or expense [53]. In this study, the decrease in protein, lipid, and glycogen content of *R. dominica* treated with *Thymus* essential oils was probably due to the pest’s greater need for these energy sources to synthesize esterase enzymes. Indeed, based on the multiple modes of action of essential oils, including the inhibition of acetylcholinesterase, adenosine triphosphatases (ATPases) and glutathione-*S*-transferase activity and disruption in gamma-aminobutyric acid (GABArs) and octopamine receptors [63], along with reduction in energy reserves and digestive enzyme activity and antinutritional effects established in this study, the chances of the pest resistance will be low. In other words, the co-evolution of herbivorous insects and plants continues, and according to this study, *Thymus* essential oils can overcome the herbivorous insect *R. dominica*.

The nutritional indices, including consumption index (CI), efficiency of conversion of ingested food (ECI), relative consumption rate (RCR), and relative growth rate (RGR), of *R. dominica* adults treated with *Thymus* essential oils particularly *T. kotschyanus* essential oil were changed. The feeding deterrence index (FDI) of the both LC_30_ and LC_50_ of essential oils from 20.41% to 66.11% was also considerable. The nutritional indices describe the utilization of diets and or effectiveness of digestion that how the insect can convert foods or their own biomass [54]. Decreases in nutritional indices of insects can lead to growth retardation due to reduce in energy resources, fertility, and fecundity, and even life stage longevity [64]. In agreement with our findings, ECI, RCR, and RGR of *R. dominica* were decreased by the essential oils of *Eucalyptus dundasii* Maiden [65], *E. floribendi* Hugel ex Endi [66], *Ferula assa-foetida* L., *Juglans regia* L., and *Pelargonium hortorum* Bailey [67]. The antinutritional effects of *Thymus* essential oils were also investigated by the some recent study. For example, a reduction in ECI, RCR, and RGR along with 57.83% FDI by 2 µL/mg food of the larvae of Indian meal moth (*Plodia interpunctella* (Hübner)) treated by *T. daenensis* Celak were reported [68]. In another study, ECI, RCR, and RGR of the larvae of Colorado potato beetle (*Leptinotarsa decemlineata* (Say)) treated with *T. daenensis* essential oil, rich in thymol (72.3%), carvacrol (7.1%), and γ-terpinene (4.8%), were significantly decreased compared to the control group [69]. Furthermore, 14 ppm of essential oil caused about 40% FDI for treated larvae. Reduction of nutritional indices of the mentioned pests by *T. daenensis* essential oil is in line with the results of this study. However, differences in FDI values may be due to differences in insects tested, essential oils used, and their chemical components. Strong antifeedant effects of thymol-rich chemotype essential oils of *T. vulgaris* and *T. zygis* Loefl. ex L. (with FDI of 74.9% and 72.3%, respectively) against the larvae of Egyptian cotton leaf worm *Spodoptera littoralis* (Boisduval) were reported by Valcárcel et al. [70]. They also indicated that the terpenes α-pinene, limonene, linalool, 1,8-cineole, camphor, *p*-cymene, carvacrol, thymol, and menthone had significant antifeedant effects, in which α-pinene, carvacrol, and thymol with high FDI (67.3%, 55.8%, and 52.4%, respectively) were more effective. Almost all above-mentioned terpenes were recognized in *Thymus* essential oils investigated in the present study, and accordingly, it can be said that their antinutritional effects may be related with such terpenes. Menthone, despite in low percentage, existed only in *T. vulgaris* and *T. kotschyanus* essential oils (0.4% and 0.5%, respectively), which can have an effect on the higher FDI of these essential oils than others. Furthermore, the more FDI of *T. vulgaris* than *T. kotschyanus* may be related to the high percentage of thymol in this oil which was not identified in *T. kotschyanus*.

## 4. Materials and Methods

### 4.1. Plant Materials and Extraction of Essential Oils

The *Thymus eriocalyx*, *T. fallas*, *T. kotschyanus*, and *T. vulgaris* plants were collected from Malayer (34°17′50″ N 48°49′23″ E Hamedan province), Saqqez (36°12′20″ N 46°13′19″ E Kurdistan province), Sardabeh (38°17′01″ N 48°02′07″ E Ardabil province), and Ardabil (38°14′46″ N 48°14′26″ E Ardabil province) of Iran, respectively (Geographic Coordinates from Google Maps). *Thymus* species were recognized according to the keys reported by Jamzad [71]. The voucher specimens were deposited with their scientific names at the Department of Plant Sciences, Moghan College of Agriculture and Natural Resources, University of Mohaghegh Ardabili, Ardabil, Iran. Five cm aerial parts including stems, leaves and flowers from the top of each separate plant were air dried in the room temperature. The specimens were pulverized with an electric grinder. The extraction of essential oils was carried out using a Clevenger apparatus with a 2000-mL flask, 100 g of the each pulverized specimen, and 1200 mL distilled water over a 3-h period. Anhydrous sodium sulphate was used to remove water from obtained essential oils, which were reserved in glass containers covered by aluminum foil and stored in refrigerator at 4 °C.

### 4.2. Chemical Profile of the Essential Oils

Gas chromatographic—mass spectral (GC-MS) analysis of the essential oils was carried out as previously described [72]: Agilent 7890B GC, Agilent 5977A MS (Santa Clara, CA, USA); MS EI mode (electron energy = 70 eV, scan range = 10–550 amu, and scan rate = 3.99 scans/sec); HP-5ms fused-silica GC (30 m length × 0.25 mm diameter × 0.25 μm film thickness); He carrier gas (column head pressure = 53.1 kPa, flow rate = 1.0 mL/min); inlet temperature = 280 °C, interface temperature = 280 °C; GC oven temperature program (50 °C, hold for 1 min, ramp 8 °C/min to 100 °C, ramp 6 °C/min to 110 °C, hold for 1 min, 6 °C/min to 310 °C, hold for 1 min); 1 μL injections of 1% *w*/*v* solution of each sample in methanol, splitless mode. The essential oil compositions were determined by comparison of MS fragmentation patterns and GC retention indices (RI) with those reported in the databases [20,21,22].

### 4.3. Insect Rearing

A colony of *R. dominica* was obtained from the Department of Plant Protection, University of Mohaghegh Ardabili, Iran. Insect rearing was performed in cylindrical plastic containers, in which the openings were covered by a mesh cloth for aeriation. The adults were released on crushed wheat (200 g, Aftab cultivar) into the containers which were kept in a growth chamber at 28 ± 1 °C, 60 ± 5% relative humidity, and 14:10 h light: dark conditions. The synchronized 1-d-old adults were selected for the experiments.

### 4.4. Fumigant Toxicity

Twenty 1-d-old adults were transferred to glass containers (140 mL) as fumigant chambers. The calculated concentrations (78.54, 86.61, 99.46, 114.24, 131.16, and 149.94 µL/L for *T. eriocalyx*; 64.26, 73.76, 86.25, 100.39, 116.88, and 135.66 µL/L for *T. kotschyanus*; 85.68, 97.68, 107.1, 117.38, 128.74, and 142.8 µL/L for *T. fallax*, and 92.82, 101.82, 114.24, 125.24, 140.52, and 157.08 µL/L for *T. vulgaris*) were poured on filter paper discs (3 cm in diameter) using a micropipette. Based on the preliminary tests, the lower and upper concentrations causing about 25 to 75% mortality of treated insects, respectively, were selected, and the others were calculated via logarithmic intervals [73]. The filter papers treated with each essential oil concentration were affixed to the inner surface of the glass container lids using adhesive tape. The lids of glass containers were rendered impermeable to air using parafilm. Mortality of treated adult insects was recorded after 24, 48, and 72 h exposure times. All steps were considered for control groups without adding essential oil concentrations or any solvent, and the experiments were repeated three times.

### 4.5. Biochemical Assays

LC_30_ (lethal concentration to kill 30% of treated insets) and LC_50_ (lethal concentration to kill 50% of treated insets) values calculated from 24 h-fumigant toxicity were selected for the biochemical assays: 84.16 and 111.16 µL/L for *T. eriocalyx*, 85.37 and 105.64 µL/L for *T. fallax*, 75.57 and 101.45 µL/L for *T. kotschyanus*, and 103.24 and 125.28 µL/L for *T. vulgaris*. 

Bovine serum albumin and Fast Blue RR salt were purchased from Roche Co. (Germany) and Merck Co. (Germany), respectively. Other biochemical materials from reagents to enzyme substrates were purchased from Sigma Chemical Co. (USA). Treatments by the essential oil concentrations and the control were repeated three times in all biochemical assays.

#### 4.5.1. Effect of Essential Oils on Energy Reserves

The whole bodies of 100 1-d-old adults treated with LC_30_ and LC_50_ of essential oils for 24 h were homogenized using a hand-held glass homogenizer in in 250 μL of sodium phosphate buffer (0.04 M, pH 7.0) at 4 °C, and the resulting homogeneous mixtures were centrifuged at 10,000 rpm for 10 min (Sigma 1–14 K refrigerated centrifuge, USA). The lipid content of adult insects was measured using vanillin reagent according to the method described by Van Handel [74]. The protein content was estimated using bovine serum albumin as a standard, according to the Bradford method [75]. The enthrone reagent was used to estimate the glycogen content [76], and the absorption was recorded spectrophotometrically at 626 nm (Unico, UV/Vis 2100, Dayton, NJ, USA).

#### 4.5.2. Effect of Essential Oils on Detoxifying Esterase Enzymes

The whole bodies of fifty alive adults treated with essential oil concentrations were homogenized in 250 μL of sodium phosphate buffer (0.04 M, pH 7.0) on ice. The homogeneous mixtures were centrifuged at 10,000 rpm for 15 min, and the resulting supernatant was separated and refrigerated at −20 °C as an enzymatic extract. The activity of α- and β-esterases was measured by the following procedures [77]: The enzyme extract (12.5 μL) was mixed with 112.5 μL of sodium phosphate buffer and 50 μL of α-naphthyl acetate (α-NA) and β-naphthyl acetate (β-NA), respectively, for α- and β-esterase. The mixtures were incubated at 30 °C for 15 min, and then50 mg of Fast Blue RR salt in 50 mL of sodium phosphate buffer (0.075 M, pH 7.0) was added. The absorbance was recorded for α-NA and β-NA during 7 min intervals at 450 and 540 nm, respectively, using a microplate reader (ELIZA-Reader, Anthos 2020, UK).

#### 4.5.3. Effect of Essential Oils on Digestive Amylase and Protease Enzymes

To measure the amylase enzyme activity, 20 μL of the enzyme extract, 500 μL of acetate buffer (50 mM, pH 6.0), and 40 μL of 1% soluble starch were reacted at 37 °C within 30 min. Then, 100 μL of dinitro salicylic acid reagent (DNSA: Sigma Chemical Co., St. Louis, USA) was added, boiled in a water bath for 10 min, and centrifuged at 15,000 rpm at 4 °C for 5 min. Three replications were considered for each treatment and blank and the absorbance was recorded at 540 nm. The amount of enzyme required to produce 1 mg of maltose at 37 °C was considered as amylase activity unit [78].

To measure the protease activity, azocasein substrate was used based on the method of Elpidina et al. [79]. Twenty μL of enzyme extract was mixed with 80 μL of azocasein solution (1.5%) in 2-morpholinoethanesulfonic acid buffer (MES: 50 mM, pH 6.0), and incubated at 37 °C for 50 min. The enzymatic reaction was blocked by adding 100 μL of 30% trichloroacetic acid (TCA). The unhydrolyzed azocasein was then precipitated by refrigeration at 4 °C within 0.5 h, and the mixture was centrifuged at 15,000 rpm for 10 min. One hundred μL of the supernatant was added to 100 μL of 2 M sodium hydroxide and the absorbance was recorded at 440 nm. Three replications were considered for each treatment and blank. In the blank, the enzyme extract was added to the reaction mixture after adding 30% TCA. A change in optical density per milligram protein per minute was defined as the protease activity unit.

### 4.6. Effect of Essential Oils on Nutritional Indices

To evaluate the antinutritional effects of *Thymus* essential oils against *R. dominica* adults, 200 1-d-old adults were separately treated with LC_30_ and LC_50_ of the essential oils. For each concentration, the alive insects were divided into 7 groups (10 insects for each). The insects were transferred to 6-cm Petri dishes containing 3 g of crushed wheat grains (Aftab cultivar). After two weeks, the food consumption, the adults’ weight, and the weight gain of insect pest were determined. The initial and remaining food and the fed and unfed adults were separately weighed after two weeks. To determine the percentage of dry weight of food and *R. dominica*, twenty samples were weighed, dried in an oven (60 °C for 48 h) and then re-weighed (Sartorius AG Germany GCA803S, d = 0.001 ct). Nutritional indices, including Consumption Index (CI), Relative Consumption Rate (RCR), Relative Growth Rate (RGR), and Efficiency of Conversion of Ingested food (ECI) were calculated based on the formulae described by Waldbauer [80]: CI = F/A, RCR = F/TA, RGR = G/TA, and ECI= G/F, Where, A = mean dry weight of insect during feeding period (mg), G = dry weight gain of insect during feeding period (mg), F = dry weight of food eaten (mg), and T = feeding period (day). Feeding deterrence index (FDI) was calculated as (FDI) (%) = [(C-T)/C] × 100, where C = the mean weight of food eaten in control and T = the mean weight of food eaten in treatment [81].

### 4.7. Statistical Analysis

For the agglomerative hierarchical cluster (AHC) analysis, the chemical compositions of 20 *T. kotschyanus* essential oils from Iran were treated as operational taxonomic units (OTUs). The percentage compositions of 11 major components (*p*-cymene, 1,8-cineole, linalool, borneol, α-terpineol, geraniol, geranial, thymol, carvacrol, α-terpinyl acetate, and geranyl acetate) were used to evaluate the phytochemical relationship between the Iranian *T. kotschyanus* essential oils by AHC analysis using the XLSTAT software, version 2018.1.1.62926 (Addinsoft, Paris, France). Pearson correlation was used as a measure of similarity, and the unweighted pair-group method with arithmetic average (UPGMA) was used to define the clusters.

Statistical software SPSS version 16.0 (IBM, Chicago, IL, USA) was used to analyze the insecticidal experiments. The normality of mortality data was checked by Kolmogorov-Smirnov test. Probit analysis was used to calculate the Lethal Concentrations (LC) and their 95% confidence limits along with concentration-mortality regression lines details. The data heterogeneity was also evaluated by χ² test. Data obtained from all insecticidal bioassays were subjected to analyses of variance, and the comparison of means were performed by the least significant difference (LSD) test.

## 5. Conclusions

The use of plant-derived essential oils to manage stored-product insect pests has been highly considered due to their rapid degradation in the environment and lower impact on non-target organisms than conventional chemicals. According to this study, the cosmopolitan stored-grain insect pest *R. dominica* is susceptible to the essential oils isolated from aerial parts of wild-grown *Thymus* species, including *T. eriocalyx*, *T. fallax*, *T. kotschyanus*, and *T. vulgaris*. Although the activity of detoxifying enzymes α- and β-esterase was augmented in the adults treated by the essential oils, along with significant fumigant toxicity, the terpene-rich essential oils showed promising sublethal effects from biochemical disturbances to antinutritional activities. The energy resources was affected by the essential oils: The protein content decreased by all essential oils, the glycogen content decreased by *T. kotschyanus* and *T. vulgaris* essential oils, and the lipid content significantly reduced by *T. fallax*, *T. eriocalyx* and *T. vulgaris* essential oils. The amylase and protease digestive enzyme activities were also affected by the essential oils except *T. vulgaris*. Furthermore, nutritional indices of insect pest especially RGR and RCR were affected by the essential oils so that they caused considerable FDI from 20.4% to 61.1%. It was also found that *Thymus* essential oils are complex mixture of terpenic compound that are probably responsible agents in the multiple modes of action of essential oils. However, further research is recommended to investigate the side effects of these essential oils on stored products and beneficial non-target insects and to evaluate the pesticidal effects of their compounds to other insect pests along with effects on human health. Furthermore, the introduction of durable formulations against environmental factors may be effective in their application.

## Figures and Tables

**Table 1:** Chemical composition (%) of essential oils isolated from aerial parts of *Thymus eriocalyx, T. fallax, T. kotschyanus,* and *T. vulgaris* essential oils.

RI_calc_	RI_db_	Compound	*T. eriocalyx*	*T. fallax*	*T. kotschyanus*	*T. vulgaris*
921	924	α-Thujene	0.2	0.1	tr	tr
934	933	α-Pinene	1.6	3.1	2.9	0.6
949	948	Camphene	2.4	0.8	0.1	0.3
953	951	3-Methylcyclohexanone	---	---	0.1	---
970	969	Sabinene	---	0.1	---	---
975	975	β-Pinene	1.2	0.4	2.2	0.1
980	979	3-Octanone	---	0.4	---	---
981	987	1-Octen-3-ol	---	---	---	0.1
988	987	Myrcene	1.1	1.0	1.8	0.5
994	988	Dehydro-1,8-cineole	---	0.1	---	---
998	996	3-Octanol	---	0.2	---	---
1012	1014	α-Terpinene	0.2	0.4	0.1	---
1025	1025	*p*-Cymene	4.8	7.5	2.2	3.4
1030	1029	Limonene	1.4	---	5.3	0.8
1031	1031	1,8-Cineole	7.4	5.4	5.7	7.6
1046	1044	(*E*)-β-Ocimene	0.1	0.1	1.1	---
1055	1054	γ-Terpinene	0.5	0.2	0.9	---
1064	1066	*cis*-Sabinene hydrate	2.1	---	---	0.2
1067	1067	*cis*-Linalool oxide (furanoid)	---	0.6	0.2	---
1079	1080	Diallyl disulfide	---	---	0.3	---
1084	1084	trans-Linalool oxide (furanoid)	---	2.8	---	---
1092	1098	trans-Sabinene hydrate	3.0	---	---	---
1101	1099	Linalool	3.2	15.4	9.5	4.1
1110	1110	1-Octen-3-yl acetate	---	---	0.2	---
1125	1118	*cis*-*p*-Menth-2-en-1-ol	---	0.3	---	---
1126	1128	*allo*-Ocimene	---	---	0.2	---
1140	1135	*trans*-Pinocarveol	---	0.4	0.2	---
1147	1146	Camphor	6.5	2.0	0.3	0.7
1157	1160	*iso*-Borneol	---	0.7	0.2	0.3
1158	1158	*iso*-Menthone	---	---	0.4	0.5
1170	1169	Borneol	5.7	0.6	0.6	2.0
1175	1174	Terpinen-4-ol	3.2	6.0	0.4	1.3
1188	1185	α-Terpineol	4.8	4.7	11.4	7.7
1190	1191	Hexyl butanoate	---	---	0.2	---
1209	1211	Octyl acetate	---	---	0.2	---
1226	---	Unidentified	---	---	1.2	---
1230	1227	Nerol	---	0.7	5.6	4.3
1232	1235	Thymyl methyl ether	0.3	0.5	---	2.3
1237	1241	Carvacryl methyl ether	1.3	0.9	---	---
1252	1248	Geraniol	0.4	2.6	---	18.7
1254	1254	Linalyl acetate	---	0.6	7.9	---
1261	1264	Geranial	0.2	---	---	---
1279	1282	(*E*)-Anethole	---	---	2.3	4.2
1279	1282	Bornyl acetate	---	2.6	---	---
1289	1289	Thymol	8.6	12.1	---	9.6
1289	1287	*iso*-Bornyl acetate	---	0.6	---	---
1301	1299	Carvacrol	19.3	2.4	1.6	9.7
1349	1343	α-Terpinyl acetate	4.0	6.3	18.9	0.2
1355	1356	Eugenol	---	0.1	---	---
1361	1359	Neryl acetate	0.3	0.4	1.8	6.3
1368	1370	Carvacryl acetate	---	0.2	---	---
1372	1374	α-Copaene	---	0.1	---	---
1381	1379	Geranyl acetate	2.0	1.7	2.6	---
1383	1387	β-Bourbonene	---	0.4	0.1	---
1408	1408	(Z)-β-Caryophyllene	0.3	---	---	---
1419	1418	(*E*)-β-Caryophyllene	3.7	1.9	0.3	1.2
1429	1430	β-Copaene	tr	0.1	---	---
1439	1439	Aromadendrene	0.1	0.3	---	---
1453	1454	α-Humulene	0.2	0.1	---	---
1460	1458	*allo*-Aromadendrene	0.1	0.1	---	---
1469	1476	Geranyl propanoate	tr	---	---	---
1475	1478	γ-Muurolene	0.1	0.1	---	0.4
1479	1479	*ar*-Curcumene	---	0.5	---	---
1481	1484	Germacrene D	0.1	---	---	---
1482	1487	(*E*)-β-Ionone	---	---	---	0.2
1487	1489	β-Selinene	0.1	---	0.2	---
1491	1496	Valencene	0.1	---	---	---
1492	1495	γ-Amorphene	---	---	---	0.3
1494	1496	Viridiflorene	---	0.2	---	---
1496	1498	α-Selinene	0.2	---	---	---
1499	1500	α-Muurolene	tr	---	---	0.2
1505	1505	β-Bisabolene	0.5	0.4	1.3	0.5
1508	1514	Geranyl isobutyrate	0.1	---	---	0.2
1513	1513	γ-Cadinene	tr	0.4	---	0.6
1518	1517	Myristicin	---	---	3.8	---
1522	1523	δ-Cadinene	0.1	0.6	---	1.0
1540	1541	(*E*)-α-Bisabolene	1.7	0.1	---	---
1551	1555	Elemicin	---	---	0.6	---
1553	1552	(Z)-Caryophyllene oxide	---	0.4	---	---
1558	1562	Geranyl butyrate	0.2	0.2	---	0.7
1561	1563	(*E*)-Nerolidol	0.2	---	0.1	0.3
1564	1564	8-Acetoxycarvotanacetone	---	---	0.3	---
1579	1578	Spathulenol	---	1.8	0.9	0.7
1585	1583	Caryophyllene oxide	3.8	2.8	0.3	0.3
1593	1595	6-Methoxyelemicin	---	---	0.4	---
1598	1594	Carotol	---	---	0.2	---
1599	1601	Geranyl 2-methylbutyrate	0.1	0.2	---	0.6
1603	1599	*anti*,*anti*,*anti*-Helifolen-12-al B	---	0.1	---	---
1604	1607	Geranyl isovalerate	---	---	---	0.1
1605	1602	Ledol	---	0.1	---	---
1612	1608	Humulene epoxide II	0.1	0.5	---	---
1618	1619	1,10-di-*epi*-Cubenol	---	0.1	0.1	0.1
1630	1631	Caryophylla-4(12),8(13)-dien-5α-ol	0.1	0.3	0.1	---
1634	1634	*cis*-Cadin-4-en-7-ol	---	---	0.1	---
1640	1640	Caryophylla-4(12),8(13)-dien-5β-ol	0.4	---	---	0.1
1641	1640	*τ*-Cadinol	---	0.4	tr	0.9
1648	1646	Himachal-2-en-7β-ol	0.1	---	---	---
1656	1654	*α*-Cadinol	---	0.2	0.2	0.3
1661	1666	14-Hydroxy-9-*epi*-(Z)-caryophyllene	---	0.4	---	0.2
1664	1668	*ar*-Turmerone	---	1.1	---	---
1666	1665	Intermedeol	1.6	---	---	---
1673	1669	14-Hydroxy-9-*epi*-(*E*)-caryophyllene	0.3	0.5	---	0.4
1682	1677	Apiole	---	---	0.8	---
1684	1685	α-Bisabolol	0.1	---	---	---
1688	1687	Eudesma-4(15),7-dien-1β-ol	0.1	0.1	0.2	---
1691	1700	Amorpha-4,9-dien-2-ol	---	---	---	0.3
1698	1699	Curlone B	---	0.1	---	---
1751	1749	Geranyl hexanoate	0.1	---	---	---
1845	1845	Phytone	---	---	0.1	0.3
1862	1860	Platambin	---	---	0.1	---
1900	1900	Nonadecane	---	---	---	0.4
1928	1921	Methyl palmitate	---	---	---	0.2
1973	1960	Palmitic acid	---	---	---	0.8
2309	2310	Isopimarol	---	---	0.3	---
2742	2747	Geranyl palmitate	---	---	---	0.6
		Monoterpene hydrocarbons	13.4	13.6	16.8	5.6
		Oxygenated monoterpenoids	72.8	71.0	67.6	77.7
		Sesquiterpene hydrocarbons	7.1	5.5	2.0	4.2
		Oxygenated sesquiterpenoids	6.6	8.9	2.1	3.6
		Diterpenoids	0.0	0.0	0.3	0.0
		Phenylpropanoids	0.0	0.1	7.9	4.2
		Others	0.0	0.6	1.0	2.0
		Total identified	100.0	99.8	97.7	97.2

RI_calc_ = Retention index calculated with respect to a homologous series of *n*-alkanes on a HP-5ms column. RI_db_ = Retention index from the databases [20,21,22]. tr = trace (<0.05%). --- = not detected.

**Table 2:** Probit analyses of data obtained from of the fumigant toxicity of *Thymus eriocalyx*, *T. fallax*, *T. kotschyanus*, and *T. vulgaris* essential oils against *Rhyzopertha dominica* adults.

Essential oil	Time (h)	LC_50_ with 95% Confidence Limits (µL/L)	LC_90_ with 95% Confidence Limits (µL/L)	RP ^a^	χ² (df = 4)	Slope ± SE	Sig. ^b^	*r* ^2^
*T. eriocalyx*	24	111.2 (105.2–127.3)	219.4 (188.7–280.4)	1.127	2.856	4.341 ± 0.553	0.582	0.958
	48	107.5 (102.4–112.9)	191.9 (171.5–227.7)	1.165	2.695	5.092 ± 0.565	0.610	0.972
	72	106.0 (101.3–110.9)	180.9 (164.0–208.8)	1.182	3.431	5.523 ± 0.573	0.488	0.970
*T. fallax*	24	105.6 (100.6–110.2)	177.6 (160.4–210.4)	1.186	1.010	5.666 ± 0.736	0.908	0.984
	48	104.0 (99.67–107.9)	162.1 (150.0–182.2)	1.205	1.530	6.658 ± 0.756	0.821	0.982
	72	103.3 (99.09–107.0)	157.0 (146.4–174.1)	1.213	2.215	7.045 ± 0.764	0.696	0.977
*T. kotschyanus*	24	101.5 (95.7–108.5)	208.4 (177.6–268.2)	1.234	1.150	4.099 ± 0.490	0.886	0.984
	48	97.77 (92.52–103.8)	191.9 (167.0–237.2)	1.281	1.675	4.377 ± 0.493	0.795	0.980
	72	95.49 (90.82–100.6)	174.5 (155.7–206.3)	1.312	1.898	4.892 ± 0.502	0.755	0.982
*T. vulgaris*	24	125.3 (120.5–130.7)	201.0 (182.1–233.9)	1.000	1.371	6.240 ± 0.706	0.849	0.983
	48	121.8 (117.7–126.1)	182.5 (168.4–203.1)	1.029	1.066	7.297 ± 0.723	0.900	0.990
	72	120.2 (116.4–124.1)	174.7 (163.7–191.4)	1.042	1.356	7.888 ± 0.736	0.852	0.989

^a^ Relative potency (RP) = 24 h-LC_50_ value of *T. vulgaris* essential oil/another LC_50_ value. ^b^ Since the significance level is greater than 0.05, no heterogeneity factor is used in the calculation of confidence limits. The number of tested insects is 420 each time.

**Table 3:** The effects of LC_30_ and LC_50_ values of *Thymus eriocalyx*, *T. fallax*, *T. kotschyanus*, and *T. vulgaris* essential oils on energy reserves (µg/adult) (mean ± SE) of *Rhyzopertha dominica* adults.

Treatment	Protein Content		Glycogen Content		Lipid Content	
	LC_30_	LC_50_	LC_30_	LC_50_	LC_30_	LC_50_
Control	126.17 ± 2.19 a	129.83 ± 2.89 a	57.22 ± 5.37 a	55.28 ± 6.08 a	6.93 ± 0.97 a	6.60 ± 0.83 a
*T. eriocalyx*	111.83 ± 4.60 a	105.17 ± 4.51 b	55.11 ± 3.04 a	50.22 ± 6.04 ab	6.67 ± 0.24 a	4.73 ± 0.64 bc
*T. fallax*	113.16 ± 7.90 a	93.67 ± 11.23 b	55.50 ± 8.69 a	49.01 ± 6.86 ab	5.47 ± 0.35 a	3.67 ± 0.29 c
*T. kotschyanus*	121.67 ± 6.34 a	105.33 ± 1.09 b	43.56 ± 1.59 a	36.90 ± 1.68 b	6.13 ± 0.41 a	5.53 ± 0.35 ab
*T. vulgaris*	122.17 ± 3.63 a	105.83 ± 4.04 b	46.06 ± 6.65 a	36.33 ± 0.58 b	6.07 ± 0.87 a	4.40 ± 0.50 bc
ANOVA (df = 4, 10)	*F* = 1.36 *p* = 0.316	*F* = 5.09 *p* = 0.017	*F* = 1.20 *p* = 0.367	*F* = 2.91 *p* = 0.048	*F* = 0.79 *p* = 0.555	*F* = 4.050 *p* = 0.033

Mean values in a column followed by different lowercase letter are significantly different on the basis of ANOVA with LSD test (*p* < 0.05).

**Table 4:** The effects of LC_30_ and LC_50_ values of *Thymus eriocalyx*, *T. fallax*, *T. kotschyanus*, and *T. vulgaris* essential oils on esterase enzymes activity (µmol/min/mg protein) (mean ± SE) of *Rhyzopertha dominica* adults.

Treatment	α-Esterase Activity		β-Esterase Activity	
	LC_30_	LC_50_	LC_30_	LC_50_
Control	0.041± 0.001 c	0.038± 0.0007 c	0.119 ± 0.005 c	0.123 ± 0.004 c
*T. eriocalyx*	0.053 ± 0.004 b	0.054 ± 0.002 b	0.143 ± 0.003 b	0.146 ± 0.002 b
*T. fallax*	0.052 ± 0.004 b	0.056 ± 0.003 b	0.164 ± 0.003 a	0.165 ± 0.001 a
*T. kotschyanus*	0.068 ± 0.001 a	0.069 ± 0.001 a	0.140 ± 0.007 b	0.148 ± 0.004 b
*T. vulgaris*	0.070 ± 0.002 a	0.072 ± 0.001 a	0.162 ± 0.004 a	0.167 ± 0.002 a
ANOVA (df = 4, 10)	F = 18.74 *p* < 0.001	*F* = 47.09 *p* < 0.001	F = 16.27 *p* < 0.001	F = 32.04 *p* < 0.001

Mean values in a column followed by different lowercase letter are significantly different on the basis of ANOVA with LSD test (*p* < 0.05).

**Table 5:** The effects of LC_30_ and LC_50_ values of *Thymus eriocalyx*, *T. fallax*, *T. kotschyanus*, and *T. vulgaris* essential oils on amylolytic (mg maltose/min/individual) and proteolytic (mU/individual) enzyme activity (mean ± SE) of *Rhyzopertha dominica* adults.

Treatment	Amylolytic Activity		Proteolytic Activity	
	LC_30_	LC_50_	LC_30_	LC_50_
Control	0.400 ± 0.026 a	0.410 ± 0.026 a	0.121 ± 0.014 a	0.113 ± 0.032 a
*T. eriocalyx*	0.353 ± 0.033 a	0.283 ± 0.023 bc	0.105 ± 0.030 a	0.059 ± 0.013 bc
*T. fallax*	0.317 ± 0.072 a	0.227 ± 0.026 c	0.102 ± 0.017 a	0.057 ± 0.014 bc
*T. kotschyanus*	0.307 ± 0.063 a	0.220 ± 0.029 c	0.099 ± 0.016 a	0.048 ± 0.004 c
*T. vulgaris*	0.380 ± 0.045 a	0.347 ± 0.043 ab	0.110 ± 0.024 a	0.101 ± 0.024 ab
ANOVA (df = 4, 10)	*F* = 0.61 *p* = 0.664	*F* = 6.71 *p* = 0.007	*F* = 0.16 *p* = 0.953	*F* = 3.73 *p* = 0.042

Mean values in a column followed by different lowercase letter are significantly different on the basis of ANOVA with LSD test (*p* < 0.05).

**Table 6:** The effects of LC_30_ and LC_50_ values of *Thymus eriocalyx*, *T. fallax*, *T. kotschyanus*, and *T. vulgaris* essential oils on nutritional indices (mean ± SE) of *Rhyzopertha dominica* adults.

Nutritional Indices	Concentration (µL/L)	Control	*T. eriocalyx*	*T. fallax*	*T. kotschyanus*	*T. vulgaris*	ANOVA (df = 4, 30)
CI (mg/mg)	LC_30_	7.69 ± 0.82 a	5.17 ± 0.89 b	5.87 ± 1.01 ab	4.81 ± 0.67 b	5.09 ± 0.89 b	*F* = 2.15: *p* = 0.039
	LC_50_	8.04 ± 0.59 a	5.34 ± 0.92 bc	6.54 ± 1.03 ab	4.16 ± 0.58 c	4.37 ± 0.72 bc	*F* = 4.21: *p* = 0.008
ECI (%)	LC_30_	3.06 ± 0.18 a	2.92 ± 0.54 a	1.54 ± 0.31 b	1.39 ± 0.23 b	2.96 ± 0.57 a	*F* = 4.34: *p* = 0.007
	LC_50_	2.83 ± 0.21 a	2.55 ± 0.21 a	1.06 ± 0.09 b	1.33 ± 0.35 b	2.64 ± 0.24 a	*F* = 12.12: *p* < 0.001
RCR (mg/mg/day)	LC_30_	0.57 ± 0.06 a	0.37 ± 0.06 b	0.42 ± 0.07 ab	0.32 ± 0.0 7 b	0.36 ± 0.06 b	*F* = 2.15: *p* = 0.049
	LC_50_	0.56 ± 0.04 a	0.33 ± 0.06 b	0.41 ± 0.06 b	0.30 ± 0.04 b	0.27 ± 0.05 b	*F* = 5.87: *p* = 0.001
RGR (mg/mg/day)	LC_30_	0.017 ± 0.001 a	0.009 ± 0.002 b	0.006 ± 0.002 bc	0.004 ± 0.001 c	0.009 ± 0.001 b	*F* = 10.18: *p* < 0.001
	LC_50_	0.016 ± 0.007 a	0.008 ± 0.001 b	0.004 ± 0.0007 cd	0.004 ± 0.0008 d	0.007 ± 0.0008 bc	*F* = 32.12: *p* < 0.001
FDI (%)	LC_30_	-	36.73	20.41	40.82	44.90	-
	LC_50_	-	50.00	37.04	57.41	61.11	-

Mean values in a row followed by dissimilar lowercase letters are significantly different on the basis of ANOVA with LSD test (*p* < 0.05). CI = consumption index; ECI = Efficiency of conversion of ingested food; RCR = Relative consumption rate; RGR = Relative growth rate; FDI = feeding deterrence index.

## Data Availability

The data that support the findings of this study are available upon reasonable request.
